# High attack rate in a Tong Lau house outbreak of COVID-19 with subdivided units in Hong Kong

**DOI:** 10.1098/rsfs.2021.0063

**Published:** 2022-02-11

**Authors:** Qun Wang, David Christopher Lung, Pak-To Chan, Wei Jia, Chung-Hin Dung, Te Miao, Jianxiang Huang, Wenzhao Chen, Zixuan Wang, Kai-Ming Leung, Pengcheng Xu, Zhang Lin, Daniel Wong, Herman Tse, Sally Cheuk Ying Wong, Garnet Kwan-Yue Choi, Kelvin Kai-Wang To, Vincent Chi-Chung Cheng, Kwok-Yung Yuen, Yuguo Li

**Affiliations:** ^1^ Department of Mechanical Engineering, The University of Hong Kong, Hong Kong SAR, People's Republic of China; ^2^ Department of Urban Planning and Design, Faculty of Architecture, The University of Hong Kong, Hong Kong SAR, People's Republic of China; ^3^ Estates office, The University of Hong Kong, Hong Kong SAR, People's Republic of China; ^4^ Department of Microbiology, Li Ka Shing Faculty of Medicine, The University of Hong Kong, Hong Kong SAR, People's Republic of China; ^5^ Department of Pathology, Hong Kong Children's Hospital, Hong Kong SAR, People's Republic of China; ^6^ Department of Pathology, Queen Elizabeth Hospital, Hong Kong SAR, People's Republic of China; ^7^ Environmental Protection Department, Hong Kong SAR, People's Republic of China; ^8^ Institute of Applied Mathematics, Academy of Mathematics and Systems Sciences, Chinese Academy of Sciences, Beijing, People's Republic of China; ^9^ Division of Building Science and Technology, City University of Hong Kong, Hong Kong SAR, People's Republic of China; ^10^ Department of Microbiology, Queen Mary Hospital, Hong Kong SAR, People's Republic of China

**Keywords:** COVID-19, SARS-CoV-2, poor housing, drainage system, building ventilation

## Abstract

Poor housing conditions are known to be associated with infectious diseases such as high Coronavirus disease 2019 (COVID-19) incidences. Transmission causes of severe acute respiratory syndrome coronavirus 2 (SARS-CoV-2) in poor housing conditions can be complex. An understanding of the exact mechanism of transmission can help to pinpoint contributing environmental issues. Here, we investigated a Hong Kong COVID-19 outbreak in early 2021 in four traditional Tong Lau houses with subdivided units. There are more than 80 subdivided units of less than 20 m^2^ floor area each on average. With a total of 34 confirmed COVID-19 cases, the outbreak had an attack rate of 25.4%, being one of the highest attack rates observed in Hong Kong, and ranked among the highest attack rates in reported outbreaks internationally. Tracer gas leakage and decay measurements were performed in the drainage system and in the subdivided units to determine the transport of infectious aerosols by the owner-modified sophisticated wastewater drainage pipe networks and the poor ventilation conditions in some subdivided units. The results show that the outbreak was probably due to multiple transmission routes, i.e. by the drainage pipe spread of stack aerosols, which is enhanced by poor ventilation in the subdivided units.

## 1. Introduction

Underprivileged communities have been hit the hardest in the Coronavirus disease 2019 (COVID-19) pandemic [1,2], where patients from poor housing conditions were associated with higher COVID-19 incidence and mortality [3]. The exact environmental conditions that can lead to the transmission of severe acute respiratory syndrome coronavirus 2 (SARS-CoV-2) in poor housing conditions remain unknown. As an example, Hong Kong has around 200 000 people living in so-called subdivided units (also referred to as subdivided flats) of 6–10 m^2^ or even less [4,5]. These subdivided units are formed by dividing a small flat into several tiny units for rental. Each subdivided unit has its own toilet-cum-bathroom for privacy [6]. In Hong Kong, these subdivided units are usually built in the so-called Tong Lau (literally means ‘Chinese building’) before the 1960s, which is like a low-rise tenement with one flat on each floor. These flats generally share an entrance stairway access. Tong Lau is one of the three main types of housing, and other two are public (rental) housing (44.8% of the population) and private housing (54.6% of the population) in Hong Kong. Tong Lau is known to be a more suboptimal environment than the other two types of housing [7], which is made worse by the increasing number of subdivided units following the housing shortage. The subdivided units also experience poor air quality due to overcrowding and insufficient ventilation [4,8]. Access to these subdivided units for field study is generally very difficult.

**Figure 1.**
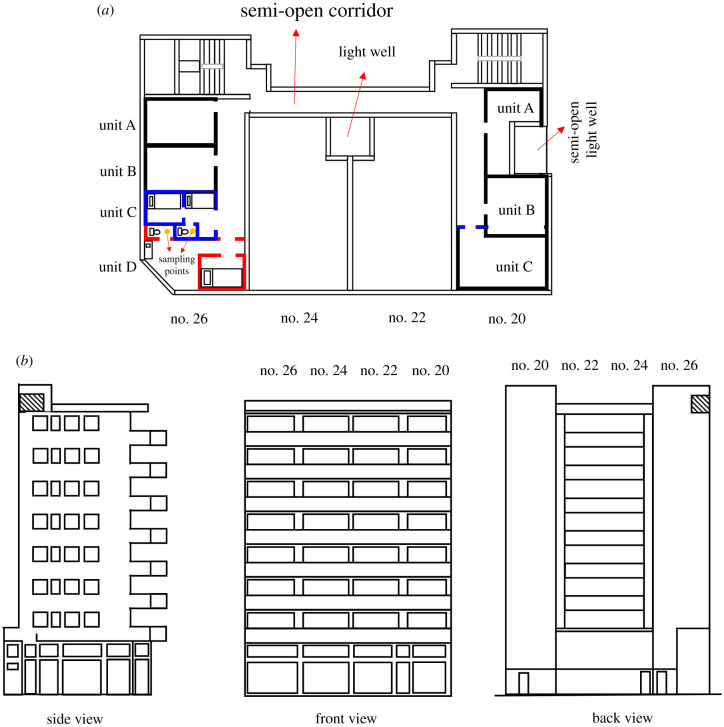
The 5th-floor plan, and design of the four Tong Lau houses. (*a*) Floor plan and subdivided units on the 5th floor: the orange circles represent the sampling points in Units 5C (no. 26) and 5D (no. 26); the subdivided unit's design at no. 20 Reclamation Street was assumed according to the location of the door of each unit, as we did not have access to the interior. (*b*) Building design recreated according to the drawings in 1962–1965. Unit 5C (no. 26) refers to the subdivided unit C of the 5th-floor flat in no. 26 Reclamation Street. Two Google Earth images of the building are shown in the electronic supplementary material, figure S4.

A large COVID-19 outbreak occurred in early January 2021 in four Tong Lau houses, involving more than 80 subdivided units with a total of 34 confirmed COVID-19 cases (figure 1). The four ‘houses’, in a single building with common semi-open corridors and one stairwell, are referred to as nos. 20, 22, 24 and 26 Reclamation Street, built in 1962–1965. These are typical Tong Lau houses in the highly populated Yau Ma Tei area in Kowloon. Each Tong Lau house consists of seven storeys with shops occupying the ground floor. In the original design, one flat has a floor area of 62.5 m^2^ on each of the upper floors. These flats were subdivided into 3–5 tiny units on average on each storey for rental. On each floor, the four flats of the corresponding ‘houses’ (nos. 20, 22, 24 and 26) are inter-connected by a common corridor with one side of the corridor opening to the outdoor. In each original flat, the subdivided units shared a narrow common corridor with a width of around 0.9 m.

The 20–26 Reclamation Street outbreak occurred during a period when a surge of COVID-19 cases was confirmed in its neighbourhood area. Between 1 and 20 January 2021, a total of 162 COVID-19 cases were identified within an area of 56 buildings including the above-mentioned four Tong Lau houses in Jordan, Kowloon. This area was designated as a ‘restricted area’ by the Government for COVID-19 intervention, with the subsequent extension of compulsory testing of all residents or persons present for more than 2 h within the ‘core area’ bounded by Temple Street to its east, Ning Po Street to its south, Reclamation Street to its west and Pak Hoi Street to its north, within which are about 70 buildings in total. The buildings in the area are relatively old, with a predominance of these traditional Tong Lau houses and subdivided units are common. To our knowledge, there have not been any investigations of COVID-19 transmission in a similar overcrowded environment of a Tong Lau house with subdivided units. With the largest number of 34 confirmed cases, and the highest attack rates, the 20–26 Reclamation Street Tong Lau outbreak (referred to as 20–26 Tong Lau outbreak in the rest of the text) offered a unique opportunity for an environmental study to investigate complex environmental factors of the outbreak in poor housing.

The residents from the four Tong Lau houses were sent to quarantine centres for two-week isolation from 15 January 2021. Following a disinfection campaign in the buildings and an approval by the Centre for Health Protection (CHP), our research team gained access to carry out on-site tracer gas studies with the consent of residents during the last 2 days of the quarantine period, which was preceded by two site visits 2 days earlier.

## 2. Methods

We first obtained the dates of symptom onset and sub-unit addresses of the confirmed COVID-19 cases from the CHP, Department of Health and local media (table 1). We also obtained the building data, original site plan (figure 1) and plumbing layout from the Buildings Department of Hong Kong. The owner or residents subdivided each flat into smaller units for rental. The exact time of subdivision is unknown; however, the interior conditions of the subdivided units were found to be decent and clean. Each subdivided unit has its own toilet-cum-bathroom, which is confirmed by our visits to five units. The subdivided unit floor plan, and the new drainage system drawings are not available. We had access to the common semi-open corridors of the four Tong Lau houses, some shared interior corridors of subdivided units, and only 5 subdivided units. According to our site visits, each flat is subdivided into 3–4 units (subdivided units) with two flats being subdivided into 6 or 7 units (table 2 and figure 1). An approximate floor plan of typical subdivided units on the 5th floor of no. 26 Reclamation Street is shown in figure 1*a*. These subdivided units are numbered, e.g. unit 3A (no. 20) is the subdivided unit A of the 3rd-floor flat in no. 20 Reclamation Street.

**Table 1.** Description of the 34 infected cases according to street number and flat number.

street no.	unit no.	gender (age)	symptom onset date	confirmation date	case no.^a^	notes
20	3A	M (56)	asymptomatic	15 Jan	20-3A-1	
	4B	M (50)	asymptomatic	10 Jan	20-4B-1	Central Kowloon Route—Central Tunnel cluster
		F (47)	05 Jan	12 Jan	20-4B-2	
		M (28)	05 Jan	12 Jan	20-4B-3	
	5B	F (42)	09 Jan	12 Jan	20-5B-1	
		F (22)	10 Jan	12 Jan	20-5B-2	
		M (41)	07 Jan	13 Jan	20-5B-3	
22	1B	F (70)	06 Jan	12 Jan	22-1B-1	
		M (75)	asymptomatic	12 Jan	22-1B-2	
24	5F	M (37)	10 Jan	14 Jan	24-5F-1	
		F (0)	13 Jan	15 Jan	24-5F-2	
		F (28)	14 Jan	15 Jan	24-5F-3	
	7B	F (32)	asymptomatic	17 Jan	24-7B-1	
26	1D	F (33)	08 Jan	12 Jan	26-1D-1	
	4C	F (50)	30 Dec	11 Jan	26-4C-1	
	5B	F (61)	11 Jan	13 Jan	26-5B-1	
		M (31)	05 Jan	13 Jan	26-5B-2	
		F (62)	05 Jan	13 Jan	26-5B-3	
	5C	F (40)	11 Jan	13 Jan	26-5C-1	
		M (18)	asymptomatic	15 Jan	26-5C-2	
		M (20)	13 Jan	15 Jan	26-5C-3	
	5D	M (30)	02 Jan	04 Jan	26-5D-1	Tseung Kwan O—Lam Tin Tunnel construction site cluster
	6A	F (47)	27 Dec	30 Dec	26-6A-1	
		M (47)	29 Dec	31 Dec	26-6A-2	
	6B	F (32)	asymptomatic	12 Jan	26-6B-1	
		F (26)	asymptomatic	12 Jan	26-6B-2	
		F (31)	asymptomatic	13 Jan	26-6B-3	
	6C	M (77)	01 Jan	08 Jan	26-6C-1	
		F (19)	asymptomatic	11 Jan	26-6C-2	
		F (68)	asymptomatic	11 Jan	26-6C-3	
	6D	F (46)	09 Jan	11 Jan	26-6D-1	
	7B	F (34)	asymptomatic	11 Jan	26-7B-1	
		F (44)	asymptomatic	11 Jan	26-7B-2	
		F (33)	09 Jan	11 Jan	26-7B-3	

^a^All COVID-19 confirmed cases are given a number by CHP. Here, a new case code is given to each case so that no individual can be identified.

**Table 2.** The number of subdivided units in the four Tong Lau houses.

floor/street no.	no. 20	no. 22	no. 24	no. 26
1st floor	3	3	unknown	7
2nd floor	unknown	3	2	4
3rd floor	3	3	4	4
4th floor	unknown	3	unknown	4
5th floor	3	unknown	6	6
6th floor	unknown	unknown	unknown	4
7th floor	unknown	3	3	4

Tracer gas (SF_6_) measurements were conducted on 27–28 January 2021 just before the residents returned from their quarantine centres. Tang *et al*. [9] and Zhang *et al*. [10] found that when the airflow removal dominates, a tracer gas can mimic well the dispersion of airborne particles (particularly exhaled droplet nuclei) smaller than 5–10 μm as the effects of settling removal of these fine particles are relatively small. We monitored the tracer gas concentration in five infected units, i.e. Unit 1D (no. 26), Unit 5C (no. 26), Unit 5D (no. 26), Unit 3A (no. 20) and Unit 5B (no. 20), façades and roof vents by using a 24-channel multipoint sampler, a photoacoustic gas monitor (Innova 1412i and 1409, LumaSense Technologies, Ballerup, Denmark) and a 6-channel multipoint sampler and a multi-gas monitor (type 1303 and 1302, Brüel & Kjær, Nærum, Denmark). To achieve a higher temporal resolution, we used one sampling point of each of the two sets of instruments in each flat. Before the test was started, we sealed the toilet door of units, the water basins with PVC sheets and tapes. For safety concerns, although the CHP health officers had disinfected the environment, we poured disinfectant into the sink and floor drain during the measurements, thereby the U-trap of the floor drain should be functioning during our experiment.

Three sets of tracer gas experiments were performed. First, the tracer gas was injected into the drainage stack via the toilet in Unit 5D (no. 26) or Unit 3A (no. 20) at a flow rate of 0.2 l min^−1^ with the exhaust fan on or off. Second, the tracer gas was injected into the drainage stack via the toilet in Unit 3A (no. 20) at a flow rate of 0.2 l min^−1^, while warm water was being added to the drainage pipe to simulate when a resident takes a shower under the conditions of the exhaust fan on. Third, the tracer gas was released in Unit 5D (no. 26) to study the horizontal transmission. The association between the distribution pattern of the infected units and the leaked tracer gas measurement is then discussed. Additionally, ventilation rates were estimated in Unit 5D (no. 26), while the tracer gas was released in Unit 5D (no. 26) with the exhaust fan off. The air change rate was obtained from the tracer gas decay curve.

All of the confirmed cases were admitted to a public hospital managed by the Hospital Authority in Hong Kong. Patients were isolated in an airborne isolation room, and a real-time reverse transcription polymerase chain reaction assay was performed, as described previously [11]. SARS-CoV-2 genome sequencing and subsequent sequence analysis were performed, as described previously [12], on laboratory-confirmed COVID-19 patient specimens archived in Queen Elizabeth Hospital and Queen Mary Hospital. The study protocol was approved by the Research Ethics Committee of the Kowloon Central/Kowloon East Cluster (HA.KC/KE-20-0321/ER-2) and Institutional Review Board of the University of Hong Kong/Hospital Authority Hong Kong West Cluster (UW 13-372).

## 3. Results

### 3.1. The description of the infected cases

The Tong Lau houses, 20–26 Reclamation Street, are located in one of the city's most densely populated neighbourhoods. The Tong Lau buildings at 20, 22, 24 and 26 Reclamation Street are inter-connected on each storey, e.g. all residents on the 5th floor of 22–26 share the same semi-open corridor (electronic supplementary material, figure S4), and all residents in the four Tong Lau buildings also share the same stairwell for entry. Authorities first issued a compulsory testing order for 26 Reclamation Street on 8 January 2021, and then extended it to cover 20, 22 and 24 Reclamation Street 4 days later after more cases were identified. On 15 January, the Department of Health issued a quarantine order to all other residents of the four buildings and transferred them to quarantine centres. A total of 100 residents were evacuated to the quarantine centres, and it is unknown if any other residents left the buildings before the quarantine. The total number of residents is estimated to be 134. No access to the buildings was allowed without approval during the quarantine period.

**Figure 2.**
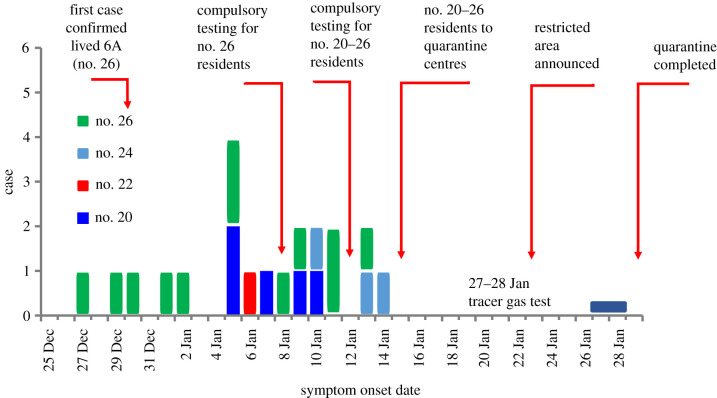
The dates of symptom onset of the 21 symptomatic cases among a total of 34 confirmed cases (13 confirmed cases were asymptomatic).

**Figure 3.**
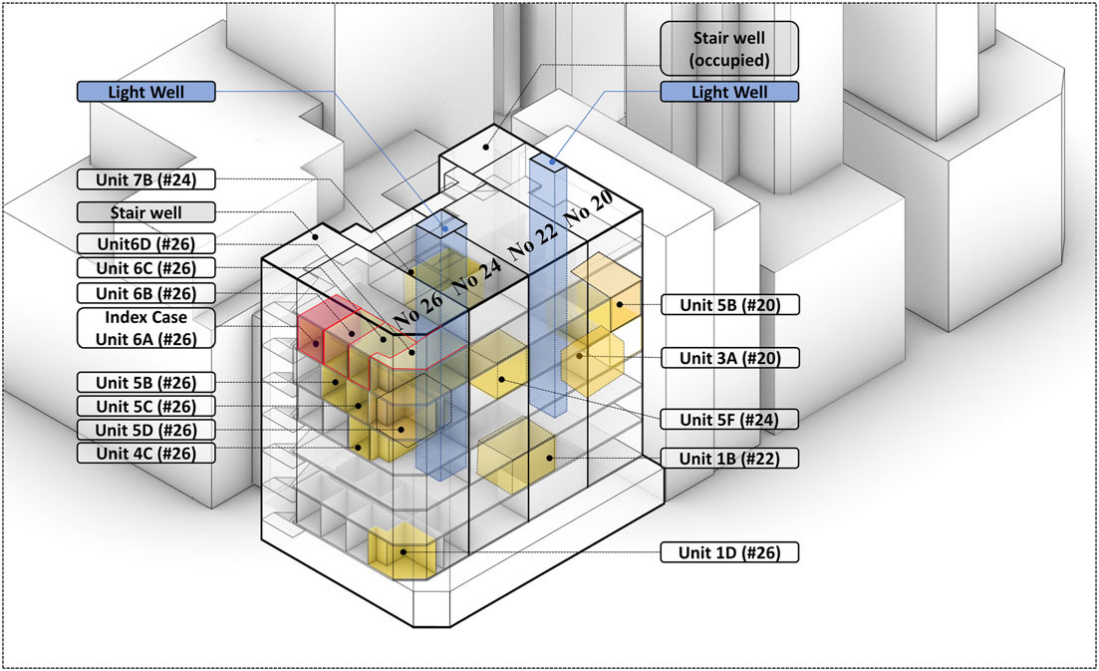
Distribution of the infected cases within the four Tong Lau houses. Although the exact unit number is correct, the exact floor plan on most floors was extrapolated from the 5th-floor plan.

Table 1 summarizes a total of 34 infected cases that occurred in 20–26 Reclamation Street. The dates of symptom onset of the 21 symptomatic cases are shown in figure 2. The index patient (case no. 26-6A-1) lived in Unit 6A (26). Four infections were linked to two construction site-related community outbreaks, the Central Kowloon Route highway and the Tseung Kwan O- Lam Tin Tunnel. Excluding these cases, a vertical distribution can be observed among these infected cases, e.g. case nos. 26-1D-1, 26-4C-1, 26-5C-1 and 26-6C-1 lived on the 1st, 4th, 5th, 6th floors, respectively, at 26 Reclamation Street and case nos. 20-5B-1 and 20-3A-1 lived on the 5th and 3rd floors, respectively, at 20 Reclamation Street (table 1 and figure 3).

**Figure 4.**
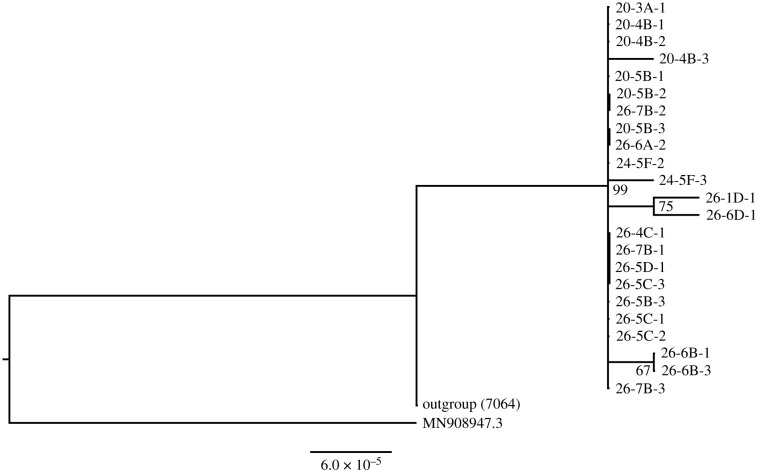
Maximum-likelihood tree of SARS-CoV-2 genome sequences from the no. 20−26 Reclamation Street cluster constructed using IQ-TREE v. 2.1.1 under the best-fit HKY + F evolutionary model. The node labels at the base of the branches indicate the branch support calculated using ultrafast bootstrap approximation with up to 5000 replicates.

SARS-CoV-2 whole-genome sequencing was successfully performed for 23 patient samples from 7 units in 20 and 14 units in 26 Reclamation Street. The maximum-likelihood phylogenetic tree of the SARS-CoV-2 genome sequence is shown in figure 4. Sequence analysis confirmed that the viral genome sequences were essentially identical in 17 cases, whereas sequences from the remaining six cases contained one to two additional nucleotide substitutions. Overall, these results support a point source in the cluster.

### 3.2. Vertical spread by drainage system

Lack of drainage system drawings and unique design of interior subdivided units causes enormous challenges for our investigation. We visited the site twice before the start of our tests to gather basic information about the buildings (figure 1; electronic supplementary material, figures S1 and S2). Our tracer gas studies focused on 20 and 26 Reclamation Street where the infected units were accessible. There was no access to any units in nos. 22–24. All non-infected units were not accessible.

A single-stack drainage system is used in 20–26 Reclamation Street, and each Tong Lau building has its own drainage system. For 26 Reclamation Street, each floor shares a long horizontal pipe around the building (shown by a blue line in the electronic supplementary material, figure S2), and all wastewater pipes of the subdivided units on the floor are connected to this horizontal pipe and then connected to the vertical stack. A hopper head seemed to be used for some wastewater discharge (electronic supplementary material, figure S2).

The index patient lived in Unit 6A (no. 26), but unfortunately, we were unable to access the unit. Instead, we had to inject the tracer gas into the drainage pipe via the toilet of Unit 5D (no. 26) which is close to the index unit. To explain the vertical cluster of confirmed cases, we monitored the tracer gas concentration in Unit 1D (no. 26) and Unit 5C (no. 26). For the horizontal cluster of confirmed cases, we monitored tracer gas concentration in Units 5D (no. 26) and 5C (no. 26). For 20 Reclamation Street, we injected tracer gas into the drainage pipe via the toilet of Unit 3A (no. 20) and monitored the tracer gas concentration in Unit 5B (no. 20) and roof vent. The sampling locations were set at around 10 cm above the floor drain of the toilet and near the roof vent.

#### 3.2.1. Test 1. Effect of exhaust fan on vertical spread

**Figure 5.**
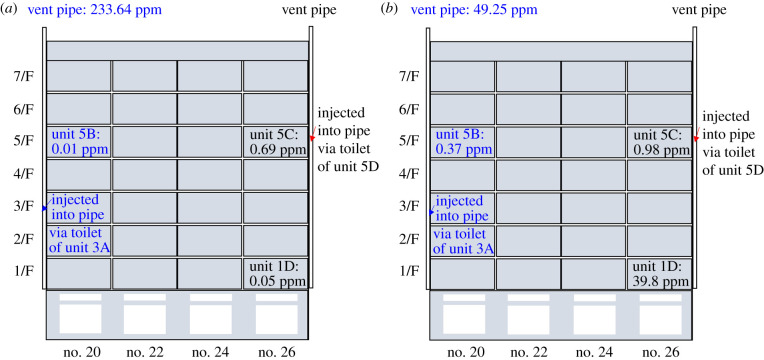
The tracer gas concentration near the floor drain of the bathroom with (*a*) exhaust fan off and (*b*) exhaust fan on. Concentrations in black and blue, respectively, represent that the tracer gas was injected into the drainage pipe via the toilets of Unit 5D (no. 26) and of Unit 3A (no. 20) during the measurement.

Figure 5 shows peak tracer gas concentration when we injected tracer gas into the drainage pipe via the toilet of Unit 5D (no. 26) and Unit 3A (no. 20), respectively. As the U-traps of the floor drains were filled with water, only Unit 5C (no. 26) captured a higher tracer gas concentration (0.69 ppm) when we injected the tracer gas into the drainage pipe via the toilet, and the concentrations in other units were sufficiently low (around 0.01–0.05 ppm) that we consider there was no obvious leakage in these units. However, when we turned on the exhaust fan in the bathroom of Unit 1D (no. 26), Unit 5C (no. 26) or Unit 5B (no. 20), the tracer gas concentration dramatically increased in all units. For example, the tracer gas concentration in Unit 1D (no. 26) increases from 0.05 to 39.8 ppm. The concentration in Unit 5B (no. 20) increases from 0.01 to 0.37 ppm.

With the exhaust fan powered on, the significant gas leakage in Unit 5C (no. 26), Unit 1D (no. 26) and Unit 5B (no. 20) is consistent with the locations where the infections were reported. Combined with the possible transmission route via the floor drain with dried-up U-trap revealed in previous studies [13,14], the major vertical spread in the vertical cluster was likely due to the drainage system.

#### 3.2.2. Test 2. Effect of adding warm water on vertical spread

When we turned on the exhaust fan of Unit 5B (no. 20) and injected tracer gas into the drainage pipe via the toilet of Unit 3A (no. 26), the peak concentration near the roof vent was around 49.25 ppm, a much lower value than 233.64 ppm from the previous test under the same condition. It may be modulated by the temperature difference in the air between the drainage system and the outdoor; therefore, we added warm water through the floor drain (the same drain was used when the residents took showers) to find out its effect on the vertical spread of tracer gas.

**Figure 6.**
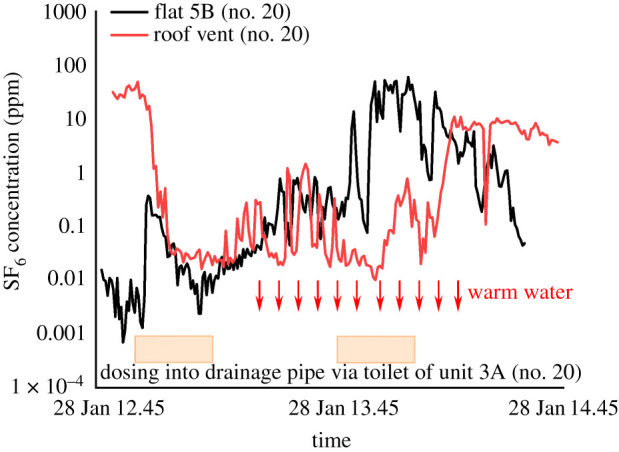
Temporal variation of tracer gas concentration when warm water was added into the drainage system via the floor drain in the bathroom of Unit 3A (no. 20). The orange rectangle represents the dosing period from the beginning to the end.

Figure 6 shows the temporal variation of concentration in the bathroom of Unit 5B (no. 20) and roof vent for the building at no. 20 Reclamation Street. An obvious fluctuating pattern of concentration was detected. The concentration dropped to around 0.02 ppm from 13.03 to 13.16, even with continuous dosing. When we started adding 2 l of 43.4°C warm water every 5 min from 13.27 through the floor drain of Unit 3A (no. 20), the concentration first dropped slightly, and then increased to around 1.1 ppm at peak. Notice this increase in concentration occurred when dosing had already been stopped. After tracer gas had been injected into the drainage pipe, a high concentration near the roof vent persisted with a small fluctuation.

Discharging warm water increased air temperature in the drainage pipes, and increased stack pressure leading to more SF_6_ detected in the flat and near the roof vent. We can imagine that in such crowded living conditions, the heat release due to shower warm water would be large enough to cause a strong buoyancy effect in the drainage stack.

### 3.3. The horizontal inter-unit transmission

**Figure 7.**
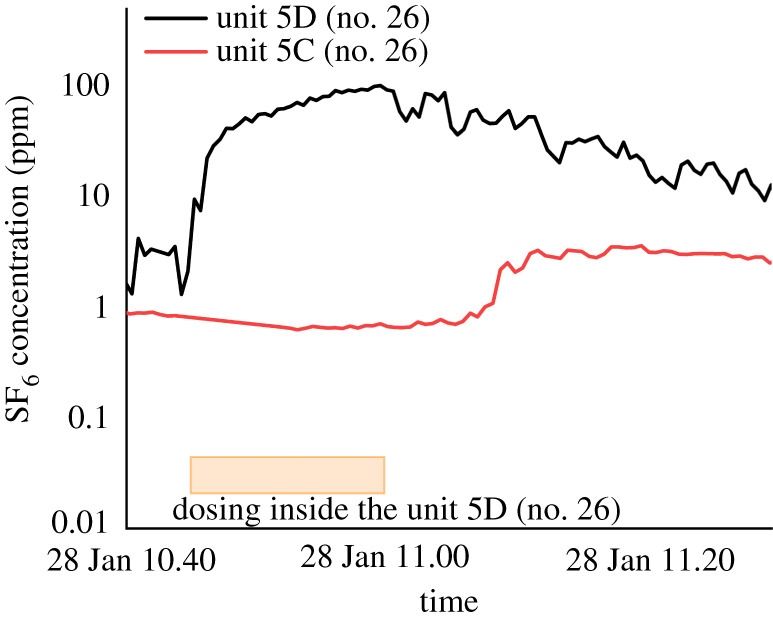
The temporal variation of tracer gas concentration in Unit 5D (no. 26) and Unit 5C (no. 26) when dosing inside the rooms of Unit 5D (no. 26). Orange rectangle denotes the dosing period. There are roughly 15 min of a delay from gas mixing in both units until detecting SF_6_ in Unit 5C.

In terms of the horizontal cluster, all units on the 6th floor at 26 Reclamation Street were infected. Three of four units on the 5th floor were also infected. Both stack aerosols from the toilet of infected/index case units and respiratory aerosols from the infected/index case units could spread into the adjacent units as these subdivided units share the same corridor (figure 1*a*). As we were releasing tracer gas in Unit 5D (no. 26), we monitored an above-background-level concentration in Unit 5C (no. 26). Figure 7 shows the detection of a tracer elevation on Unit 5C (no. 26) when releasing the tracer gas in the adjacent Unit 5D (no. 26). The doors and windows were closed, exhaust fans were off, and no one accessed the units during the measurement. This indicated that some gas escaped into the adjacent Unit 5C (no. 26) from some unnoticed leakages. The non-approved drainage pipe and kitchen exhaust ducts might be flawed due to space constraints (e.g. electronic supplementary material, figure S3). For all pipes or ducts to be directed to outside, some may have to cross the wall connecting any two units; for example, kitchen air ducts and toilet exhaust fans in Unit 5C (no. 26) were installed above the toilet of 5C (no. 26) and 5D (no. 26), hidden in ceiling panels. The air may be leaked to Unit 5C (no. 26) if there is a gap between the flexible pipe and wall or if the exhaust pipes of the subdivided units were inter-connected. The measured data thus may have revealed a potential inter-unit spread.

### 3.4. Subdivided unit ventilation rate

Under such overcrowded circumstances, the ventilation rate of the subdivided units may have also played a role in the infection transmission among these adjacent units. Therefore, we used a tracer gas concentration decay method to estimate the air exchange rate per hour (ACH) in these units by releasing the tracer gas in Unit 5D (no. 26) with windows closed. An electric fan was placed in Unit 5D (no. 26) to promote air mixing for a more uniform concentration distribution. Once a high concentration of tracer gas was detected in the dosing unit, the injection was stopped, and the concentration variation of the tracer gas in both Unit 5D (no. 26) and Unit 5C (no. 26) was continuously monitored. The sampling points were located about 1.5 m from the floor in the living room of Unit 5D (no. 26) and Unit 5C (no. 26).

**Figure 8.**
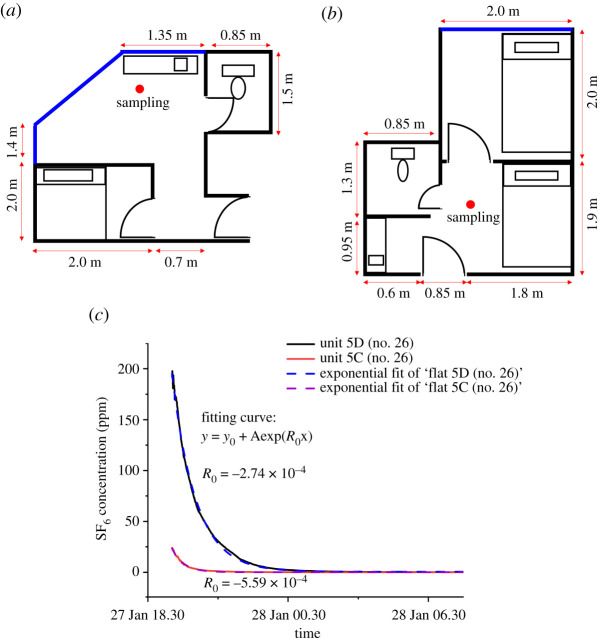
The schematic diagram of (*a*) Unit 5D (no. 26) and (*b*) Unit 5C (no. 26). The red circle represents the sampling point. (*c*) Tracer gas concentration decay and fitting curves in corresponding units during the air exchange rate experiment with tracer gas being released in Unit 5D.

Figure 8*c* shows the concentration decay in Unit 5D (no. 26) and Unit 5C (no. 26). After SF_6_ was injected for 16 min, the concentration reached a relatively steady state (approx. 190 ppm in Unit 5D (no. 26)). We also measured a relatively high concentration (approx. 24 ppm, whereas the background level on the measurement day is less than 0.1 ppm) in Unit 5C (no. 26), which indicates the tracer gas leaks to the adjacent Unit 5C (no. 26) from Unit 5D (no. 26) even with the windows and doors in both units closed (similar results in figure 7). The SF_6_ concentration decayed exponentially to nearly 0.1 ppm after 10 h for Unit 5D (no. 26) and 4 h in Unit 5C (no. 26).

The tracer decay data of Unit 5D (no. 26) were used to determine the ACH with the concentration decay method, as the tracer gas distribution should be relatively uniform in the dosing unit when the concentration reaches a steady state with the air mixing set-up. An air exchange rate of 0.99 ACH has been found in Unit 5D (no. 26). The room volume of Unit 5D (no. 26) is estimated to be 36.45 m^3^ (floor area 13.5 m^2^ × height 2.7 m), so that the estimated ‘ventilation’ rate is only 9.9 l s^−1^ for Unit 5D (no. 26), which includes transported air from neighbouring rooms. We were unable to repeat the air exchange rate measurement due to time constraints.

### 3.5. Effect of hopper head on aerosol spread

**Figure 9.**
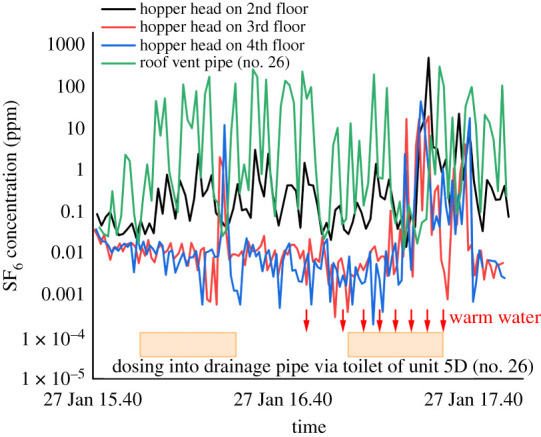
Tracer gas concentration near the hopper heads and roof vent pipe at 26 Reclamation Street. The orange rectangle represents the dosing period.

The Tong Lau houses have a special hopper head design, and its potential effect on aerosol spread was also investigated. We focused on 26 Reclamation Street, and the design of the drainage system is shown in the electronic supplementary material, figure S2. As the pipe of the hopper head and drainage stack are connected at the bottom, we injected tracer gas into the drainage stack via the toilet of Unit 5D (no. 26) and monitored the concentration near the hopper head on the 2nd, 3rd and 4th floors and the roof vent pipe. Without adding warm water to the drainage stack via the floor drain of Unit 1D (no. 26), the concentration near the hopper head on the 2nd floor subsequently increases after dosing began, although with a small fluctuation. The average concentration near the hopper head on the 2nd floor was around 0.3 ppm. For the concentration near the hopper heads on the 3rd and 4th floors, only an abrupt increase was detected at around 16.20 (figure 9); at other time slots, the concentration remained relatively low (around 0.01 ppm).

After adding 2 l of warm water at an average temperature of 42.5°C to the drainage stack, the concentration dramatically increased to a higher level (e.g. the peak concentration was 499. 8, 44.9 and 19.8 ppm on the 2nd-, 3rd- and 4th-floor hopper heads, respectively). Such a design may increase the infection risk to the surroundings.

### 3.6. Roof vent of no. 20 Reclamation Street monitoring

An unexpected phenomenon was detected on the second day of measurement. Following our plan, we conducted the tracer gas measurement by releasing the tracer gas in no. 26 Reclamation Street on the first day and no. 20 Reclamation Street on the second day. There was no tracer gas released into the drainage system of no. 20 Reclamation Street; however, at the beginning of the first test before releasing tracer gas into the drainage system of no. 20 Reclamation Street on the second day, we detected a relatively high tracer gas concentration (0.5–1.0 ppm) in its vent pipe. Although the four houses of nos. 20–26 have their individual drainage system, these drainage stacks connect at the bottom before discharge into the manhole. The tracer gas released in the drainage system at no. 26 Reclamation Street on the first day remained in the system and was detected in the vent pipe of no. 20 Reclamation Street without any further injection of the tracer gas. This also suggests that these drainage pipes were poorly ventilated while there were no residents in the buildings.

At the end of the second day, we also injected tracer gas into the drainage system of the building at no. 20 Reclamation Street and monitored the concentration in vent pipes of the buildings at no. 22 and no. 24 Reclamation Street, but none was detected (electronic supplementary material, figure S5), suggesting possible different flow direction, i.e. it was possible for no. 26 stack tracer to migrate to no. 20, but not from no. 20 to no. 22 and no. 24. The time constraint did not permit us to carry out further investigations.

## 4. Discussion

The most striking feature of this Tong Lau outbreak is the high attack rate. The estimated attack rate is 25.4% (34/134 residents). This has been one of the highest attack rates observed in Hong Kong and ranked among the highest attack rates in reported outbreaks internationally. For example, an attack rate of 67%, 36% and 22% was reported in homeless shelters [15–17], and 24% in 10 fitness centres [18]. The very high attack rate in this Tong Lau outbreak lies in the crowdedness, poor indoor ventilation without windows for many subdivided units, and unauthorized modification of the building drainage system. Our field study demonstrated satisfactory hygiene conditions in the subdivided units that we had access to, but the shared open corridor and one stairwell were blocked with poor hygiene.

The 21 confirmed symptomatic cases had symptom onset dates from 27 December 2020 to 14 January 2021, over a period of 19 days. The epidemic curve was not suggestive of a single-source infection. We used a validated back-calculation method [19,20] to estimate the exposure dates of these symptomatic cases, and three exposure periods could be identified.

**Figure 10.**
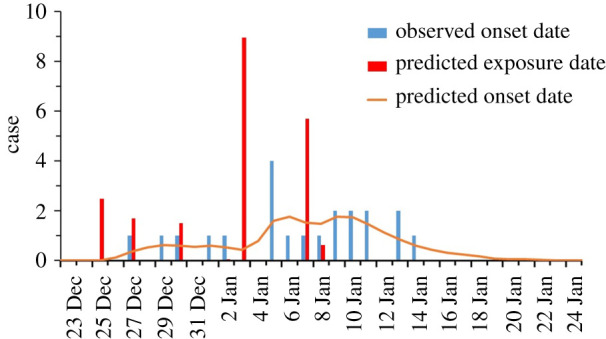
The observed dates of symptom onset and predicted dates of exposure.

Comparing figure 10 and figure 2, one may infer that the first five cases in no. 26 were exposed to the virus around 25–30 December 2020. The second exposure occurred on 3 January, which possibly led to infections from nos. 20, 22 and no. 26 and the third exposure peak occurred around 7 January. The back calculation is only indicative, as the number of cases is small and the estimated incubation period was from data in the first phase of the pandemic in the mainland [20]. From the sequencing result of the 23 residents, at least 17 are identical and the other differs by 1–2 SNP, and this is suggestive of a clonal spread originating from a single common source (figure 4). These findings suggest that the 20–26 Reclamation Street outbreak might be a propagated outbreak where a close contact transmission route is possible. While the current study does not pinpoint the exact transmission routes, it is able to identify several risk factors for transmission.

**Figure 11.**
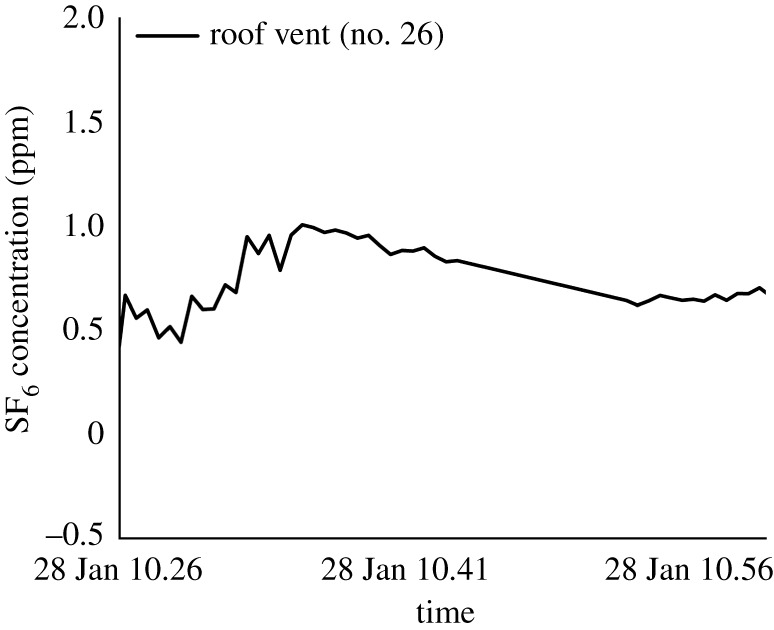
Temporal variation of tracer gas concentration in the roof vent of building no. 20 Reclamation Street on the second day.

First, tracer gas data prove the possibility of spread via the drainage system, especially in no. 26 Reclamation Street. Similar tracer gas spread tests were conducted in seven other outbreaks with a vertical pattern of the infected flats in Hong Kong and Guangzhou. In these outbreaks, the transport of the stack aerosols by the drainage pipes was shown to be likely [14]. Stack aerosols were generated within the drainage pipes and stacks when wastewater was discharged after toilet flushing or washbasin discharge. The driving force for the airflow in the drainage pipes was probably dominated by the chimney effect [14]. Our demonstration that adding warm water augmented tracer gas leakage clearly demonstrated the enhanced chimney effect. Our monitored data revealed that the stack aerosols can spread vertically in 26 Reclamation Street, but can also spread between Tong Lau houses as shown by the roof vent data (figure 11). The non-approved drainage pipe modification to accommodate for an individual toilet-cum-bathroom in each subdivided unit might have contributed to the vertical and horizontal spread via the drainage systems as shown by the tracer gas studies.

Second, the ventilation within the subdivided units was poor. Each subdivided unit is small. In no. 26 Reclamation Street, the house most affected with cases, the small 62.5 m^2^ flat on each floor was subdivided into 4 to 7 units. The ventilation conditions in these flats were expected to be very poor and were confirmed in the one unit measurement and onsite visits during our investigation. The shared narrow corridor in each flat was also not ventilated. The poorly ventilated environment could have explained why two or three infected cases were reported in some of these very small subdivided units (table 1). Our monitored data clearly showed that it is possible for the tracer gas to spread between subdivided units. Unfortunately, we were unable to identify the exact air flow route in such complex multi-zone settings and thus it was not possible to determine the effect of wind direction and thermal buoyancy. The respiratory aerosol spread is also likely due to inadequate ventilation. Airborne transmissions between units were also observed in the 2003 Amoy Garden outbreak [21] and airborne transmission of SARS-CoV-2 has gained increasing acceptability [22]. People living in close proximity such as in the same household are known to have a high attack rate. Households were found to be one of the dominant infection venues in many parts of the world [23] and crowded environments are known to facilitate the transmission of infections (e.g. [24,25]). For example, among migrant workers in Singapore, almost 93% of Singapore's COVID-19 cases were related to migrant workers in dormitories where one room may house up to 20 workers. Within the first 48 days, cases within the dormitories had surged to more than 40 000, compared with fewer than 2600 infections elsewhere in the compact city-state [24]. Fortunately, the Reclamation Street infection cluster did not lead to a similar explosive spread in Hong Kong, probably due to the swift assertive public health control measures implemented by the Government, including compulsory testing and quarantine in affected areas.

In addition to overcrowding, our site visit revealed the physical and spatial difficulties in having an adequate building wastewater drainage system in such small subdivided units. Ventilation is also limited as the toilets or kitchens are windowless. Most subdivided units in 22 and 24 Reclamation Street are also anticipated to be windowless. Hence, in addition to the crowded living environment, the poor design of the drainage system and ventilation in the studied subdivided units very likely contributed to the described massive outbreak of COVID-19 here.

There are many similar Tong Lau houses with subdivided units in Hong Kong [4,5] although the exact number is unknown. Our observations and findings revealed an urgent need to improve housing conditions in these Tong Lau houses. Proper regulation of the subdivided unit design, if it cannot be avoided, is essential.

In a developed society, these old traditional housings can be a complex issue. On the one hand, it may be possible to improve the building drainage and ventilation to minimize the infection risk and other indoor environmental issues through innovative and careful designs. The extremely small residential unit offers an opportunity for such a careful effort, e.g. using advanced analysis and design tools to maximize the system performance [26,27] as a commonly used design approach for building drainage systems may not be applicable to the unique difficulties in such extremely subdivided units, i.e. very long horizontal pipes, the need of integration into the existing vertical stack and vent pipes. The ventilation duct design also needs to be properly sized and designed as some subdivided units do not have a window. The demand for such fine design may conflict with the economic considerations of the owners as the subdivision is for rental, hence the increased cost for such delicate designs and constructions can be a major hurdle. On the other hand, there might be an option to forbid the creation of such extremely small residential units. In Hong Kong, Tong Lau residents had a significantly lower level of satisfaction towards their living environments when comparing with those in public and private housings [7]. The causality of suboptimal living environments in poor housing conditions remained to be investigated. Our study demonstrated the issue with wastewater drainage system and ventilation in subdivided units in four Tong Lau houses, with public health implications.

This study has several limitations. First, only one outbreak within four Tong Lau houses was studied. However, the observed poor drainage system design and poor ventilation conditions are likely to be similar in other subdivided units, as it is the physical space constraints in these subdivided units that limit the drainage system layout and ventilation access. Second, the experiments were carried out over a very short period. After the residents were relocated to quarantine camps, the four Tong Lau houses were subjected to thorough disinfection before the research team could perform site visits. Due to time constraints, several important experiments such as roof vent monitoring and tracer gas decay tests for ventilation rate were not repeated. Moreover, we only received permission for entry to a few subdivided units only, thus were unable to assess other potentially important elements such as if the water seals were dried out in the units of the infected cases. The complete design of the subdivided unit remained unknown, as they are likely to be owned by different landlords and built by different contractors. However, our findings in the five infected units were very illustrative of the issues associated with the complex indoor environment. Lastly, we were unable to undertake a questionnaire study to look at the social interaction among the residents, including the confirmed COVID-19 cases. However, due to the limited space within the small subdivided units, it would be highly unlikely that there will be many social gatherings in the same unit.

## 5. Conclusion

The spread of COVID-19 infection among residential buildings in this outbreak seems to be the result of a multiple-route transmission of infectious aerosols (i.e. the transport by the poor drainage system with enhanced exposure due to poor ventilation conditions, close contact due to social gathering). The adding of warm water into the drainage system, and the operation of the exhaust fan in the bathroom enhanced the suction rate of tracer gas in each unit. The horizontal cluster of confirmed COVID-19 patients among the subdivided units in one flat may be attributed to inter-unit transmission due to design flaws of the exhaust pipes. Our study offers a glimpse into the complexities of the indoor environmental issues faced by residents who live in extremely small residential units, where space constraints not only apply to people, but also in building drainage systems and ventilation. The findings in the four Tong Lau houses, which probably apply to many Tong Lau settings, may provide a reference for building designers and building regulations in Hong Kong.

## Data accessibility

Datasets are freely and publicly available on GitHub: https://github.com/Engrossm/Reclamationstreet. The data are provided in the electronic supplementary material [28].
